# Detection and genome characterization of two novel papillomaviruses and a novel polyomavirus in tree shrew (*Tupaia belangeri chinensis*) in China

**DOI:** 10.1186/s12985-019-1141-9

**Published:** 2019-03-18

**Authors:** Ping Liu, Ye Qiu, Cheng Xing, Ji-Hua Zhou, Wei-Hong Yang, Qiong Wang, Jin-Yan Li, Xi Han, Yun-Zhi Zhang, Xing-Yi Ge

**Affiliations:** 1 College of Biology, Hunan University, Changsha, 410082 China; 2 Yunnan Provincial Key Laboratory for Zoonosis Control and Prevention, Yunnan Institute of Endemic Diseases Control and Prevention, Dali, 671000 China; 3 Institute of Preventive Medicine, School of Public Health, Dali University, Dali, 671000 China

**Keywords:** Tree shrew, *Tupaia belangeri*, Papillomavirus, Polyomavirus, Tumor virus

## Abstract

**Background:**

Papillomaviruses (PVs) and polyomaviruses (PyVs) infect diverse vertebrates including human and cause a broad spectrum of outcomes from asymptomatic infection to severe disease. There has been no PV and only one PyV detected in tree shrews, though the genomic properties of tree shrews are highly similar to those of the primates.

**Methods:**

Swab and organ samples of tree shrews collected in the Yunnan Province of China, were tested by viral metagenomic analysis and random PCR to detect the presence of PVs and PyVs. By PCR amplification using specific primers, cloning, sequencing and assembling, genomes of two PVs and one PyV were identified in the samples.

**Results:**

Two novel PVs and a novel PyV, named tree shrew papillomavirus 1 and 2 (TbelPV1 and TbelPV2) and polyomavirus 1 (TbelPyV1) were characterized in the Chinese tree shrew (*Tupaia belangeri chinensis*). The genomes of TbelPV1, TbelPV2, and TbelPyV1 are 7410 bp, 7526 bp, and 4982 bp in size, respectively. The TbelPV1 genome contains 7 putative open-reading frames (ORFs) coding for viral proteins E1, E2, E4, E6, E7, L1, and L2; the TbelPV2 genome contains 6 ORFs coding for viral proteins E1, E2, E6, E7, L1, and L2; and the TbelPyV1 genome codes for the typical small and large T antigens of PyV, as well as the VP1, VP2, and VP3 capsid proteins. Genomic comparison and phylogenetic analysis indicated that TbelPV1 and TbelPV2 represented 2 novel PV genera of *Papillomaviridae*, and TbelPyV1 represented a new species of genus *Alphapolyomavirus*. Our epidemiologic study indicated that TbelPV1 and TbelPV2 were both detected in oral swabs, while TbelPyV1 was detected in oral swabs and spleens.

**Conclusion:**

Two novel PVs (TbelPV1 and TbelPV2) and a novel PyV (TbelPyV) were discovered in tree shrews and their genomes were characterized. TbelPV1, TbelPV2, and TbelPyV1 have the highest similarity to *Human papillomavirus type 63*, *Ursus maritimus papillomavirus 1*, and *Human polyomavirus 9*, respectively. TbelPV1 and TbelPV2 only showed oral tropism, while TbelPyV1 showed oral and spleen tropism.

**Electronic supplementary material:**

The online version of this article (10.1186/s12985-019-1141-9) contains supplementary material, which is available to authorized users.

## Background

Papillomaviruses (PVs) belong to the *Papillomaviridae* family, which are non-enveloped small viruses with about 60 nm in diameter. PVs have a single molecule of circular genomic dsDNA of 5.8 to 8.6 kilobases (kb) in size. PVs infect epithelium of skin and mucosa in a variety of vertebrates, including mammals, birds, and reptiles. Recently, the *Firstpapillomavirinae* and *Secondpapillomavirinae* subfamilies in the *Papillomaviridae* family have been established. PVs in the *Firstpapillomavirinae* are classified into 53 genera, from *Alpha*- to *Zetapapillomavirus* [1–3]. In history, PV is the first tumor virus reported and was described in 1933 as an agent causing cutaneous horn-like warts in eastern cottontail rabbits [4]. PV infection leads to different outcomes, from lack of symptoms, warts, skin lesions, to carcinomas [5, 6]. Recently, 226 types of human papillomavirus (HPV) have been recorded (http://www.nordicehealth.se/hpvcenter, accessed on 2019-01-10). HPV types 16 and 18 are the main cause of cervical cancer and are considered highly carcinogenic [7].

Polyomaviruses (PyVs) belong to the *Polyomaviridae* family, which are non-enveloped small viruses with about 50 nm in diameter and have a single circular genomic dsDNA of about 5 kb in size. More than 80 species of PyVs have been nominated by the International Committee on Taxonomy of Viruses (ICTV). PyVs have been classified into 4 genera including *Alpha*-, *Beta*-, *Gamma*- and *Deltapolyomavirus*, among which, the alpha-, beta-, and deltapolyomaviruses infect mammals, whereas gammapolyomaviruses infect birds [8]. The first rodent PyV was discovered in 1953 in the house mouse and could induce epithelial tumors at multiple sites [9]. Recently, PyV infection in fish has been reported [10]. Most PyVs rarely cause significant clinical diseases in their host. However, some PyVs can cause serious diseases particularly in immunocompromised individuals, such as Merkel cell PyV (MCPyV, *Human polyomavirus 5*) causing Merkel cell cancer, BK PyV (BKPyV, *Human polyomavirus 1*) causing nephropathy and haemorrhagic cystitis, and JC PyV (JCPyV, *Human polyomavirus 2*) causing progressive multifocal leukoencephalopathy [11–16].

Tree shrews are small mammals belonging to the genus *Tupaia*, family Tupaiidae, order Scandentia. Recent studies elucidated that the genome and some basic biological properties of the tree shrew are much closer to those of primates than those of rodents, which makes the tree shrew a valuable model animal in biomedical research [17–19]. Tree shrew has been successfully used to create animal models for the studies of hepatitis B virus, hepatitis C virus, herpes simplex virus type 1 infection, and myopia, breast cancer, depression,, and others [20–22]. The Chinese tree shrew (*Tupaia belangeri chinensis*) is widely distributed in Southeast Asia, South and Southwest China. However, viruses naturally carried by *Tupaia belangeri chinensis* have been barely described. In the present study, we performed a viral metagenomic and degenerated primer-based PCR study of *Tupaia belangeri chinensis*. The epidemics of PV and PyV in *Tupaia belangeri chinensis* were revealed, and the genomes of 2 novel PVs and a novel PyV were characterized and analyzed. This is the first report of detection of PVs in tree shrew at the complete-genome level, and our result indicates that tree shrews may carry diverse PVs and PyVs. The novel viruses described here are a first step for studying the origin and evolution of PVs and PyVs in tree shrews.

## Methods

### Sample collection

From 2016 to 2017, 71 tree shrews, including 56 live ones and 15 dead ones, were captured in bushes and grass near the cropland ridge of Jianchuan County and Lufeng County of the Yunnan Province of China, during routine surveillance of pathogen control. Oral and rectal swabs were sampled from live tree shrews, and then the animals were released to nature, while dead individuals were dissected for tissue collection. Swabs were stored in cryopreservation tubes containing 1 mL of virus transport medium (VTM), and organs were kept in the same tubes, but without medium. VTM was composed of Hank’s balanced salt solution, pH 7.4, containing BSA (1%), amphotericin (15 μg ml^− 1^), penicillin (100 U ml^− 1^) and streptomycin (50 μg ml^− 1^) and filtered with the 0.22 μm filter. VTM was stored as 1 mL aliquots at − 80 °C. All samples were immediately put in liquid nitrogen for short storage, then transported to the laboratory on dry ice and stored at − 80 °C. Animal species was confirmed by sequencing the mitochondrial cytochrome b (*CytB*) gene as described previously [23].

### Viral metagenomic sequencing

To perform the viral metagenomic analysis, two libraries were constructed using pooled swabs and tissues, respectively. For swabs, totally 112 swabs from 56 living tree shrews, including 56 oral swabs and 56 rectal swabs, were pooled. For tissues, 75 tissue samples from 15 dead tree shrews were pooled. To prepare swab samples, each sample was thawed on ice, homogenized by vortexing for 30 s, and centrifuged at 13,000×g for 15 min at 4 °C. 200 μl of each supernatant were pooled together, and then filtered through a 0.45 μm filter. To prepare tissue samples, 100 mg tissue of each sample were homogenized using 1 ml phosphate buffer saline, centrifuged, pooled, and filtered. Viral particles in filtrate were enriched by ultracentrifugation, and treated with DNase and RNase to digest unprotected nucleic acid. Then viral DNA/RNA was extracted using QIAamp Viral RNA Mini Kit (Qiagen) and subjected to random PCR (rPCR) as previously described [24]. The purified rPCR products were used for library construction, and then were sequenced using the HiSeq-PE150 instrument (Illumina platform). The generated paired-end reads were debarcoded, trimmed, and *de-novo* assembled using Geneious software package (Version10.2) [25]. The assembled contigs were aligned to viral protein database using BLASTx with an E-value less than 10^− 5^.

### Viral detection and genomic sequencing

The contigs related to PV and PyV with significant E-values were used to design specific primers for screening, amplifying, and sequencing the complete genome of novel viruses in the samples. Primer sets of PV1F/R, PV2F/R, and PyVF/R were used to detect three different viruses in 112 swabs and 75 tissues, respectively. For detection PV and PyV in the single sample, the PCR was conducted in a 25 μl reaction mix containing 2.5 μl PCR reaction buffer, 5 pmol of each primer, 50 mM MgCl2, 0.5 mM dNTP, 0.1 μl Platinum Taq Enzyme (Invitrogen) and 2 μl DNA. The amplification was performed as follows: 94 °C for 4 min followed by 40 cycles consisting of 94 °C for 30 s, 50 °C for 30 s, 72 °C for 30 s, and a final extension of 72 °C for 10 min. After PCR screening and sequencing, samples showing singly positive for TbelPV1, or TbelPV2, or TbelPyV1 were chosen to amplify the complete genomes. Primer sets of PV1F1/R1, PV1F2/R2, and PV1F3/R3 were used for genomic characterization of TbelPV1; PV2F1/R1, PV2F2/R2, PV2F3/R3 for TbelPV2; and PyVF1/R1, PyVF2/R2 for TbelPyV1 (Additional file 1: Table S1). The PCR replicons were cloned into T-Vector, and at least 3 positive clones of each fragment were sequenced by Sanger method. The obtained sequences were used to assemble the three full-length viral genomes.

### Sequence analysis

The ORF Finder (National Center for Biotechnology Information) was used to predict the putative open reading frames (ORFs) and their deduced amino acid (aa) sequences. Gene sequences and encoded protein aa sequences were compared to those of known viruses with complete genomes available in GenBank. ClustalW and Geneious were used to generate and edit sequence alignment [25, 26]. The alignment data sets were used to generate the phylogenetic trees by MEGA6 under the maximum likelihood (ML) method with bootstrap values of 1000 replicates [27].

## Results

### PV and PyV in Chinese tree shrew

Two libraries, swab library and tissue library were sequenced by Illumina platform. By BLASTx searching, 15 contigs encoding proteins which are significant related to homologous proteins of PVs and PyVs were found. In detail, 10 contigs related to PV were only detected in swab library. By alignment of these contig sequences, 2 contigs with different nucleotide (nt) sequences both encode the E1 protein, indicating that 2 different PV strains were in the swab samples. These 2 PV strains were named TbelPV1 and TbelPV2.

Two long contigs almost covering the whole genome of PyV were detected in the swab library, and 3 contigs related to PyV were detected in the tissue library. These sequences of PyV contigs in the swab library and the tissue library were identical which indicates that they originate from the same PyV strain (named as TbelPyV1).

To confirm the prevalence of PV and PyV in tree shrew, the specific primer sets (Additional file 1: Table S1) designed according to the detected contig sequences of TbelPV1, TbelPV2, and TbelPyV1 were used to screen all the samples. The result showed that TbelPV1, TbelPV2, and TbelPyV1 were detected in oral swabs with 37.5, 25, and 30.4% positive rates, respectively (Table 1). Oral swabs co-infected by TbelPyV1/TbelPV1, or TbelPyV1/TbelPV2, or TbelPV1/TbelPV2, or TbelPyV1/ TbelPV1/TbelPV2 were also found. However, all the rectal swabs tested PV/PyV negative. In tissue samples, only TbelPyV1 was detected (in 7 of 15 spleens; Table 1). These data suggest that TbelPV1 and TbelPV2 exhibit oral tropism, and TbelPyV1 has oral and spleen tropism.

**Table 1:** Detection of TbelPV1, TbelPV2, and TbelPyV1 in tree shrew samples

Samples	Virus infection and co-infection rates in swabs and tissues						
	PyV1^a^	PV1^b^	PV2^c^	PyV1/PV1	PyV1/PV2	PV1/PV2	PyV1/PV1/PV2
Oral swab	21/56 (37.5%)	14/56 (25%)	17/56 (30.4%)	7/56 (12.5%)	9/56 (16.1%)	4/56 (7.1%)	2/56 (3.6%)
Rectal swab	0/56	0/56	0/56	–	–	–	–
Heart	0/15	0/15	0/15	–	–	–	–
Liver	0/15	0/15	0/15	–	–	–	–
Spleen	7/15 (46.7%)	0/15	0/15	–	–	–	–
Lung	0/15	0/15	0/15	–	–	–	–
kidney	0/15	0/15	0/15	–	–	–	–

^a^TbelPyV1; ^b^TbelPV1; ^c^TbelPV2

### Characterization of TbelPV1, TbelPV2, and TbelPyV1 genomes

To characterize the viral genomes, 3 samples singly positive for TbelPV1, or TbelPV2, or TbelPyV1 were chosen to amplify the genomic fragments (Additional file 1: Table S1). The replicons were cloned, sequenced, and assembled to obtain the complete genomes. Genomes of TbelPV1, TbelPV2, and TbelPyV1 were deposited in GenBank under the accession numbers: MK443496, MK443497, and MK443498.

### Analysis of genomes of TbelPV1 and TbelPV2

**Fig. 1:**
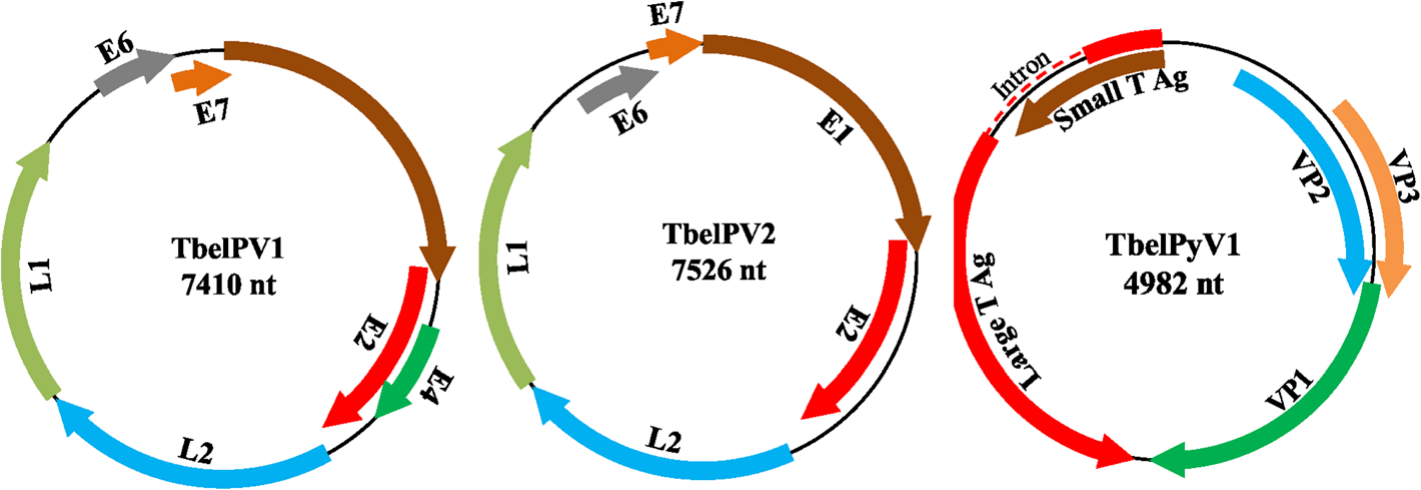
Genome organizations of the TbelPV1, TbelPV2, and TbelPyV1. The arrow indicates the predicted coding sequences and transcription direction. Dashed line indicates the LT intron

The circular genomic DNA of TbelPV1 and TbelPV2 display a length of 7410 bp and 7526 bp, with the GC content of 48.3 and 54.3%, respectively (Table 2). By BLASTx and BLASTp search, homologues of E1, E2, E4, E6, E7, L1, and L2 were found in TbelPV1 genome, and E1, E2, E6, E7, L1, and L2 were found in TbelPV2 genome. As shown in Fig. 1, the coding sequences of TbelPV1 and TbelPV2 are clockwise arranged on the same strand of the circular double stranded DNA, which is similar to other PVs. The accurate locations and lengths of the predicted ORFs, as well as the molecular weights of the corresponding proteins, are listed in Table 3.

**Table 2:** Papillomavirus genome size, GC content, and gene and protein sequence identities

Papillomavirus	GenBank number	Genome size(nt)	G + C content (%)	pairwise identities of nucleotide and amino acid sequence(nt/aa%)												
				*Tupaia belangeri chinensis* papillomavirus 1							Tupaia belangeri chinensis papillomavirus 2					
				L1	L2	E1	E2	E4	E6	E7	L1	L2	E1	E2	E6	E7
TbelPV1	MK443496	7410	48.3	/	/	/	/	/	/	/	/	/	/	/	/	/
TbelPV2	MK443497	7568	54.3	58/54	49/35	56/45	45/31	/	42/27	50/36	/	/	/	/	/	/
*Alphapapillomavirus*																
Human papillomavirus 2	NC_001352	7860	48.4	55/48	32/28	46/41	39/30	32/13	37/26	39/27	53/50	31/27	47/39	35/25	35/22	39/35
Human papillomavirus 18	NC_001357	7857	40.4	52/46	35/27	46/37	41/31	23/12	36/26	40/31	52/48	33/27	45/36	32/24	29/18	40/31
Rhesus monkey papillomavirus	NC_001678	8028	48.0	54/48	33/25	49/41	40/30	–	29/24	37/31	54/49	34/25	49/39	33/26	29/26	40/34
*Betapapillomavirus*																
Human papillomoavirus type 5	NC_001531	7746	42.4	53/49	40/35	52/44	38/29	19/8	34/25	38/35	53/50	38/35	50/44	33/23	36/27	36/36
*Chipapillomavirus*																
Canine papillomavirus 3	NC_008297	7801	51.5	55/50	35/31	51/44	38/27	–	35/24	40/29	57/56	36/30	52/44	38/26	37/22	42/32
Canine papillomavirus 4	NC_010226	7742	53.3	54/52	35/30	50/43	38/29	–	33/24	41/29	56/54	35/30	50/44	37/28	39/27	43/35
*Deltapapillomavirus*																
Bos grunniens papillomavirus type 1	JX174437	7946	44.4	54/48	30/24	47/38	39/27	34/8	28/18	27/19	52/48	28/22	46/39	32/23	32/18	31/19
*Dyodeltapapillomavirus*																
*Ursus maritimus* papillomavirus 1	NC_010739	7582	48.4	56/54	31/26	51/44	39/26	–	39/27	–	59/54	32/26	50/42	37/26	39/28	–
Feline papillomavirus type 2	EU796884	7899	53.0	55/54	35/27	51/44	34/23	–	38/27	40/34	57/58	36/29	52/47	36/25	38/28	44/32
*Dyomupapillomavirus*																
Morelia spilota papillomavirus 1	HQ262535	7048	40.9	54/50	31/26	51/43	44/33	34/14	29/21	33/25	53/54	30/25	48/44	36/27	32/26	34/24
*Dyorhopapillomavirus*																
Equine papillomavirus 2	NC_012123	7802	56.1	56/49	32/27	39/49	38/28	–	25/15	33/19	54/49	31/27	40/48	36/26	26/23	35/22
*Gammapapillomavirus*																
Human papillomavirus type 4	NC_001457	7353	38.5	53/48	33/25	51/43	43/36	41/17	37/25	37/23	52/53	34/29	48/42	36/27	36/26	36/23
*Iotapapillomavirus*																
*Mastomys natalensis* papillomavirus	NC_001605	7687	50.2	55/53	36/31	50/44	32/25	–	33/24	37/24	58/59	33/38	50/48	34/29	39/32	33/29
*Kappapapillomavirus*																
Cottontail rabbit papillomavirus	NC_001541	7868	46.4	58/56	32/26	49/44	44/36	44/12	23/12	39/31	54/51	32/28	49/43	35/29	21/10	37/28
Rabbit oral papillomavirus	NC_002232	7565	44.4	59/57	34/31	49/44	43/37	38/10	22/22	43/34	54/53	36/30	48/42	34/29	24/23	42/29
*Lambdapapillomavirus*																
Crocuta crocutapapillomavirus 1	NC_018575	8344	38.4	55/54	36/34	52/43	45/31	34/14	29/20	40/26	52/50	32/27	48/41	35/25	34/25	38/33
*Procyon lotor* papillomavirus 1	NC_007150	8170	45.9	59/59	38/37	52/46	45/38	39/15	32/20	38/26	55/53	36/30	52/46	36/28	33/25	38/38
Puma concolor papillomavirus type 1	AY904723	8321	47.2	56/55	39/37	53/47	44/31	32/21	39/23	35/26	54/50	37/31	51/44	37/27	38/27	37/30
*Mupapillomavirus*																
Human papillomavirus 1	NC_001356	7815	40.3	56/55	39/37	51/45	42/32	–	41/27	41/27	55/54	36/31	49/45	35/28	37/28	42/37
Human papillomavirus type 63	NC_001458	7348	40.4	60/59	38/36	51/44	44/34	34/12	39/29	36/28	55/56	35/32	49/43	36/26	36/25	41/34
*Zetapapillomavirus*																
Equus caballus papillomavirus type 1	NC_003748	7610	53.0	53/50	32/26	50/41	43/34	28/10	27/21	39/26	54/52	32/24	51/48	37/28	33/23	39/32

**Table 3:** Predicted open reading frames (ORFs) and noncoding regulatory regions of TbelPV1 and TbelPV2, and the molecular masses (kiloDalton) and isoelectric points (pI) of potential encoded proteins

virus	ORFs	position	length		MW kDa	pI
			nt	aa		
TbelPV1	E1	1–1824	1824	607	69.94	5.14
	E2	1775–2950	1176	391	43.64	9.83
	E4	2136–2678	543	160	20.22	6.54
	L2	2994–4628	1635	544	58.16	4.63
	L1	4639–6156	1518	505	57.07	6.55
	LCR^a^	6157-6701	545	–	–	–
	E6	6702–7121	420	139	16.26	8.07
	E7	7106–7408	303	100	11.25	3.48
TbelPV2	E1	1–1818	1818	605	68.08	6.14
	E2	1757–3148	1392	463	50.43	7.00
	L2	3261–4796	1536	511	53.81	5.53
	L1	4827–6329	1503	500	56.85	6.13
	LCR^a^	6330-6792	463	–	–	–
	E6	6793–7239	447	148	16.33	8.61
	E7	7214–7522	309	102	11.55	4.11

^a^Long control region

The E1 is the most conserved protein among PVs. The E1 protein, an ATP-dependent helicase, binds specifically to the origin of replication and is required for both the initiation and elongation of viral DNA synthesis [28]. The conserved ATP-binding sites of the ATP-dependent helicase were detected at the carboxyl terminus of E1 proteins in TbelPV1 and TbelPV2 with GNSNTGKS and GQPNTGKS sequences, respectively. E2, an auxiliary factor, is not essential for origin-dependent DNA replication, but E2 greatly enhances the ability of El to initiate DNA replication [28]. The leucine zipper domain in the E2 protein is involved in DNA-binding and dimerization of the E2 protein. The consensus sequence is L-X6-L-X6-L-X6-L in most E2 proteins of PVs. In the E2 proteins of TbelPV1 and TbelPV2, the variant sequences L-X7-L-X8-L-X6-L and L-X8-L-X6-L-X7-L were found, respectively. An E4 ORF harbored in the E2 ORF was detected in the genome of TbelPV1. The TbelPV1 E4 has a high percentage of cytosine di-, tri- and tetranucleotides, resulting in a high-proline region with content of 50%.

The E6 and E7 genes are involved in inducing transformation of infected cells. This feature permitted the mapping of the E6 and E7 as oncogenes for the high-risk PV types [7]. The zinc binding domains with conserved 36 aa sequence of C-X-X-C-X29-C-X-X-C was characterized as the zinc binding domain and the E6 and E7 proteins bind zinc through these cysteine residues [29]. For both TbelPV1 and TbelPV2, two of these domains were found in E6 protein and one in E7. The E7 proteins of TbelPV1 and TbelPV2 contain a zinc binding domain containing the L-X-C-X-E motif which is sufficient for binding of pocket protein like the retinoblastoma tumor suppressor protein [29].

A long control region (LCR) is located between the L1 ORF and the E6 ORF. The length of TbelPV1 and TbelPV2 LCRs are 545 bp and 463 bp, respectively. In the LCR of TbelPV1, 4 E2 binding sites (E2BS) were found with the consensus sequence AAC-N6-GTT. As for the LCR of TbelPV2, 4 E2BS were detected with the sequence ACC-N6-GGT.

### Gene similarities and phylogenetics of TbelPV1 and TbelPV2

The sequence similarities between TbelPV1, TbelPV2, and known PVs are shown in Table 2. The L1 is the most conserved gene among all the PVs. The identity of nt sequence in the L1 ORF between TbelPV1 and TbelPV2 was 58%. For the L1 ORF, TbelPV1 showed the highest similarity to *Human papillomavirus type 63* (HPV-63) with a maximum of 60% nt sequence identity, followed by *Rabbit oral papillomavirus* (RoPV) and *Procyon lotor papillomavirus 1* (PlPV1) both with 59% nt identities. For the L1 ORF, TbelPV2 showed the highest similarity to *Ursus maritimus papillomavirus 1* (UmPV1 with a maximum of 60% nt sequence identity, followed by *Mastomys natalensis papillomavirus* (MnPV) with 58% nt identity.

**Fig. 2:**
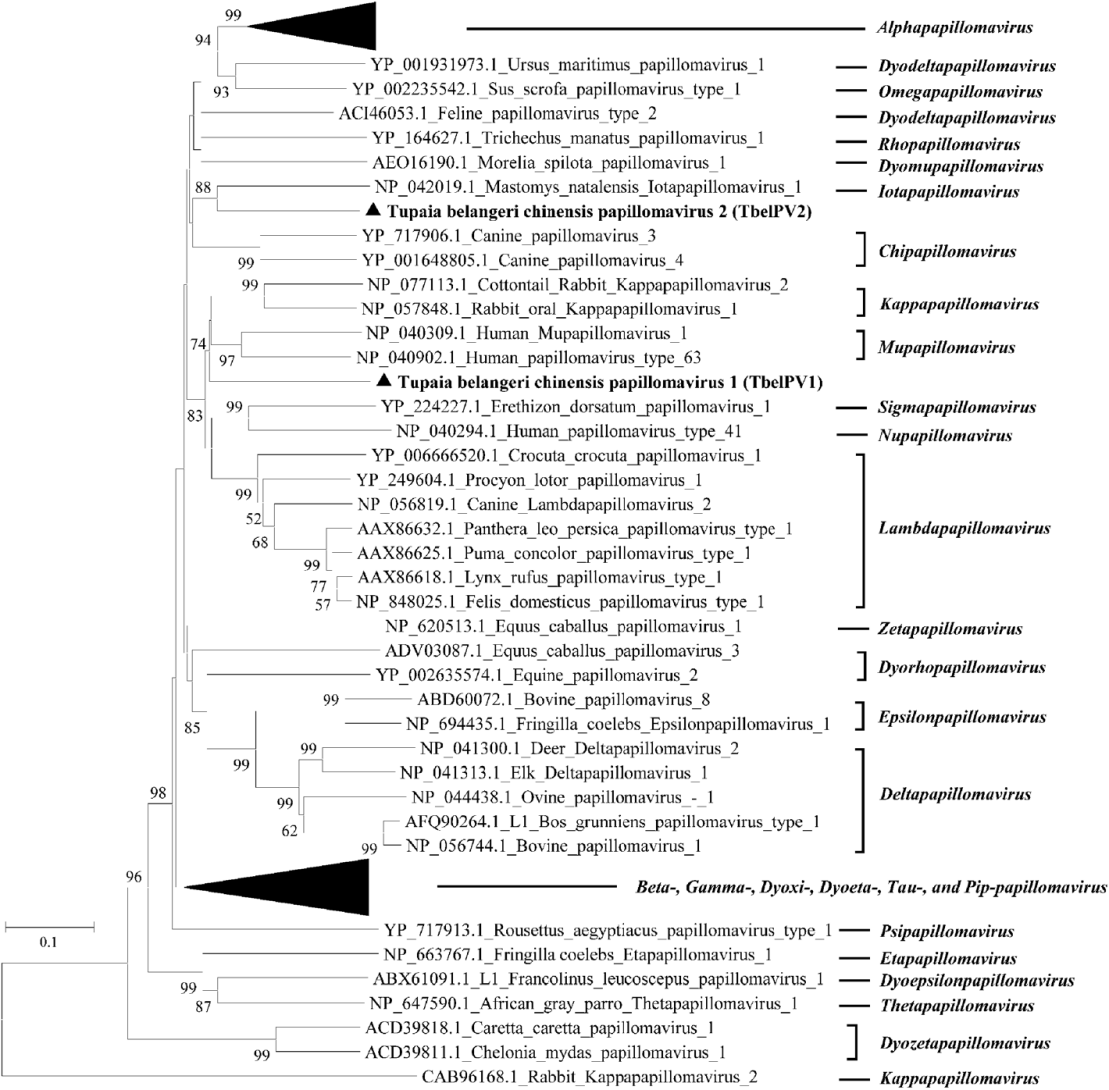
Phylogenetic analysis of TbelPV1 and TbelPV2. The tree was constructed based on alignment of complete amino acid sequences of L1 by the maximum likelihood method with 1000 bootstrap replicates. Bootstrap values above 50% are shown. For all PVs included in the tree, GenBank accession numbers, full names, and genera of are shown. TbelPV1 and TbelPV2 detected in this study are indicated in bold with solid triangle

A phylogenetic tree was constructed based on the L1 sequences of TbelPV1, TbelPV2, together with other representative animal and human PVs in the established genera. In the tree, TbelPV1 was most closely related to members of the genera *Kappapapillomavirus* and *Mupapillomavirus,* but did nest within *Kappapapillomavirus* or *Mupapillomavirus*, suggesting an early evolution. Likewise, TbelPV2 formed a separate evolutionary branch and showed a long evolutionary distance to the members of genus *Iotapapillomavirus* (Fig. 2).

### Analysis of TbelPyV1 genome

The circular genome of the TbelPyV1 was 4982 bp with overall GC content of 42.6% (Table 4). By genomic comparing and ORF searching, 5 genes and 2 noncoding regions homologous to earlier described PyVs were detected in the TbelPyV1 genome (Fig. 1 and Table 5). Two earlier genes, the small T antigen (ST) and the large T antigen (LT) were arranged on one strand, while 3 later genes including the capsid VP1, VP2, and VP3 were arranged on the opposite strand. An upstream regulatory region (URR) of 463 bp was located between the beginning of the early genes and the beginning of the late genes. A short noncoding region of 66 bp with an AT-rich region was located between LT and VP1 (Fig. 1 and Table 5). More details about the sizes and positions of predicted genes, as well as the deduced protein sequences, were listed in Table 5.

**Table 4:** Genome sizes, GC content, and gene and protein sequence identities of TbelPyV1 and other related PyVs

Polyomaviruses	GenBank numbers	Genome size(nt)	G + C content (%)	Nucleotide and amino acid sequence pairwise identities(nt/aa, %)			
				Large T antigen	Small T antigen	VP1	VP2
*Alphapolyomavirus*							
**TbelPyV1**	MK443498	4982	42.6	–	–	–	–
*Human polyomavirus 9*	NC_015150	5026	39.2	60.8/64	64.9/67.7	72/77.5	64.6/67.6
*African green monkey polyomavirus*	NC_004763	5270	40.9	59.6/61.5	64/63	69.4/76.4	62.7/67
*Pan troglodytes polyomavirus 3*	HQ385748	5309	40.9	42.4/36.9	48/39.9	52.9/55	34.7/27.7
*Pan troglodytes verus polyomavirus 2c*	HQ385749	5315	41.1	42.6/37.1	48.1/40.4	52.4/55.2	35.3/27.7
*Gorilla gorilla gorilla polyomavirus 1*	HQ385752	5300	42.2	43.2/38.2	48.9/39.4	52/54.7	35.4/28
*Pan troglodytes verus polyomavirus 1*	HQ385746	5303	40.4	43.7/38.7	49.7/41.5	53.1/56.7	36.2/28.6
*Artibeus planirostris polyomavirus 1*	JQ958887	5371	39.5	44.4/37.3	49.6/39.9	46/52.3	33.4/25.5
*Carollia perspicillata polyomavirus 1*	JQ958889	5352	42.4	44.9/37.9	48.3/39.4	42.8/49.9	32.6/25.8
*Sumatran orang-utan polyomavirus*	FN356901	5358	38.7	46.9/41.9	45.1/37	59.3/62.6	53.9/47.8
*Chimpanzee polyomavirus*	FR692334	5086	38.2	46.5/41.5	46.2/36.3	43.2/51.4	33.5/29.1
*Tupaia glis polyomavirus*	MG721015	5234	42.1	45.5/39.1	48.3/40.1	43/50.6	31.3/26.1
*Molossus molossus polyomavirus 1*	JQ958893	4903	40.9	46.1/40.4	44.9/32.3	52.8/50.6	33.1/27.1
*Artibeus planirostris polyomavirus 2*	JQ958886	5019	41.2	44.5/40.2	45.6/37	55.8/51.5	32.7/27
*Sturnira lilium polyomavirus 1*	JQ958888	5058	42.2	44.9/40.1	44.7/34.9	53.8/51.2	32.7/27.6
*Bornean orang-utan polyomavirus*	FN356900	5168	40.2	52.7/47.5	49.2/37.8	58.9/56.6	51.7/44.6
*Murine polyomavirus strain A3*	J02289	5296	47.2	42.7/37.8	42/30.9	57.2/56.9	45.6/37.6
*Hamster polyomavirus*	M26281	5366	41.5	43.5/37.3	39.9/31.8	55.6/55.9	46.2/37
*Betapolyomavirus*							
*Human polyomavirus 3*	EF127906	5040	39.4	49.3/41.5	42.4/32	36.1/24	27.8/15.1
*Murine polyomavirus 2*	EF186666	5001	40.3	48/37.7	37.4/29.3	56.8/55.4	44.6/35.4
*Mastomys natalensis polyomavirus 1*	AB588640	4899	39.9	47.6/37.7	38.3/28.4	58.2/58.4	46.6/39.1
*Myotis polyomavirus VM-2008*	NC_011310	5081	41.7	45.8/36.4	39/27.9	57.1/57.9	43/36.5
*Artibeus planirostris polyomavirus 3*	JQ958890	5187	44.2	44.4/39.8	45.9/33.7	59.5/59.1	45.1/38.4
*Pteronotus parnellii polyomavirus 1*	JQ958891	5041	38.6	45.6/38.8	43.4/34.8	57.2/57.8	43.9/37.2
*Desmodus rotundus polyomavirus 1*	JQ958892	5201	43.3	47.2/40.5	41.8/33.2	58.7/57.7	43.6/39.3
*Equus caballus polyomavirus 1*	NC_017982	4987	44.6	46.7/39.7	39/33.9	55.3/56.3	45/42.3
*Simian polyomavirus 1*	J02400	5243	40.8	44.2/41	42.9/32.8	55.5/51.8	40.5/29.5
*Human polyomavirus 1*	V01108	5153	39.5	46.1/40.6	42.6/33.3	54.8/51.5	38.4/28
*Simian polyomavirus 12*	AY614708	5230	41.2	46.2/41.6	43.1/32.8	56.4/53.1	39.8/28.9
*Deltapolyomavirus*							
*Human polyomavirus 7*	NC_014407	4952	42.9	47.5/40.8	48/40.9	36.2/27.2	31/15.6
*Human polyomavirus 6*	NC_014406	4926	42.7	46.9/39	46.3/36.8	36.3/25.7	29.3/15.1
*Human polyomavirus 10*	JX262162	4939	36.7	50.8/42.3	50.4/42.1	49.8/44.9	46.1/33.8
*Gammapolymavirus*							
*Pyrrhula pyrrhula polyomavirus 1*	DQ192571	5278	51.2	36.6/28	30.4/16.8	53.3/54.3	38.9/31.2
*Corvus monedula polyomavirus 1*	DQ192570	5079	45.7	36.7/26.9	29.6/21.6	56.5/58.3	42.4/36.4

**Table 5:** Predicted open reading frames (ORF) of TbelPyV1, and the molecular masses (kiloDalton) and isoelectric points (pI) of potential encoded proteins

ORFs	position	length		MW kDa	pI
		nt	aa		
URR^a^	1-463	–	–	–	–
VP2	464–1438	975	324	35.82	5.02
VP3	821–1438	618	205	23.96	6.71
VP1	1395–2501	1107	388	40.1	6.07
NCR^b^	2502-2567	–	–	–	–
LT	2568–4385	2058	685	78.34	6.67
	4743–4982				
ST	4413–4982	570	189	22.37	8.31

^a^Upstream regulatory region

^b^Noncoding region

The URR contains specific DNA sequences meditating the origin of PyV replication [29]. In the URR of TbelPyV1 genome, 2 copies of the perfectly consensus pentanucleotide LT binding site (GAGGC) and their reverse complement (GCCTC) were present. Between the pentanucleotide and reverse complement, a central perfect octanucleotide palindrome (TCCCTTCT and AGAAGGGA) was found. The possible sequence of core origin region of replication is **GCCTC**CGAA**GCCTC***TCCCTTCT*TTAGTC*AGAAGGGA*G**GAGGC**GA**GAGGC.**

Previously studies demonstrated that some typical elements in T antigens are necessary to fulfill infectious cycle of PyVs [30]. The predicted ST protein of TbelPyV1 is 189 aa in length and contains two CXCX2C consensus sequences (CFCITC, CFCYSC) for protein phosphatase 2A binding. The ST and LT sharing an N-terminus region of approximately 80 residues is a common feature for all PyVs. In the N-terminus of ST and LT of TbelPyV1, the highly canonical residues HPDK was found, which binds and activates the ATPase activity of host cell HSC70 [31]. In the LT of TbelPyV1, the conserved residues LXCXE (LLCSE) was detected that binds direct to the retinoblastoma family of tumor suppressor and are crucial for DNA replication [30, 32].

### Protein sequence similarities and phylogenetics of TbelPyV1

**Fig. 3:**
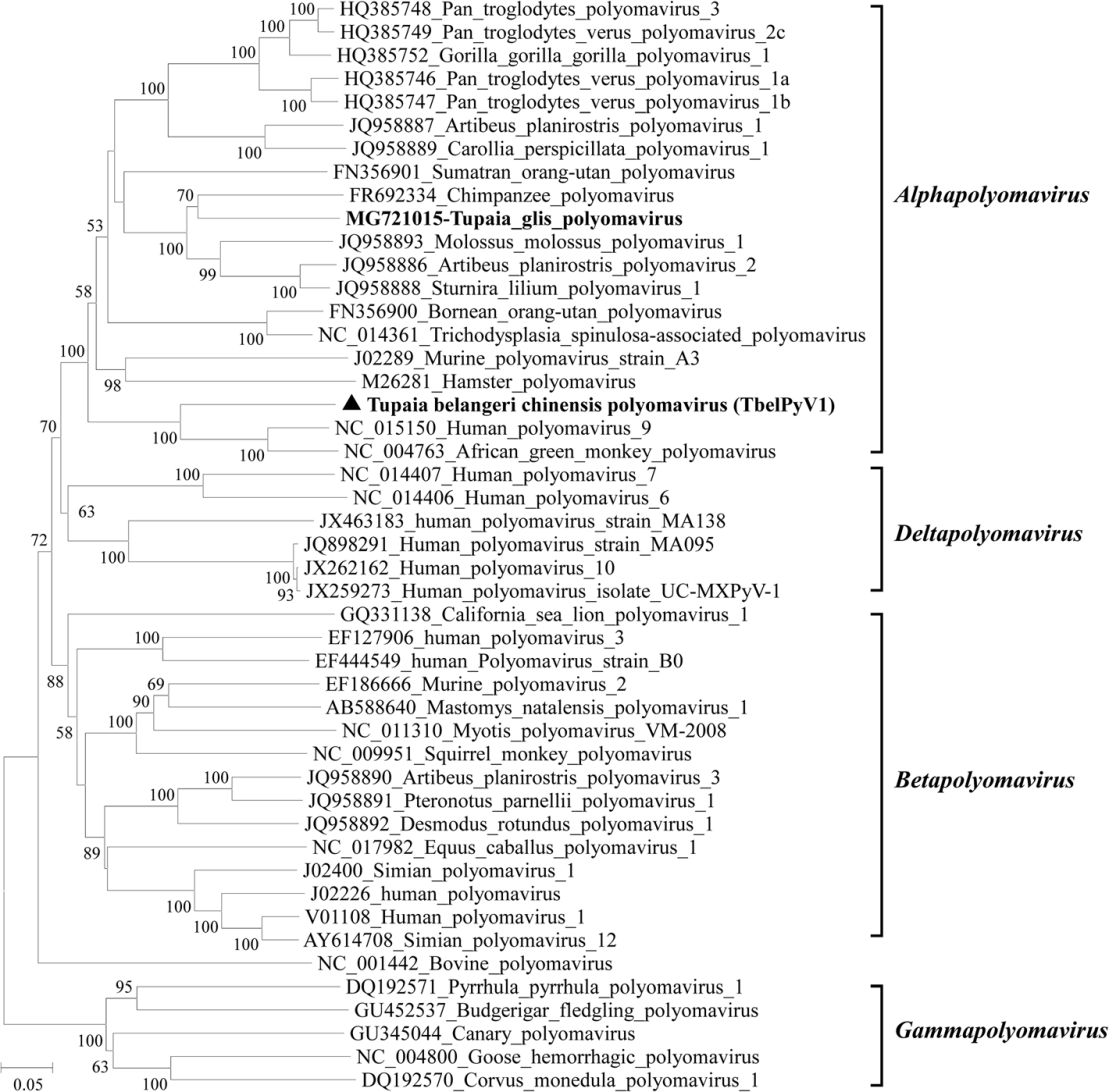
Phylogenetic analysis of TbelPyV1. The tree was constructed based on alignment of complete amino acid sequences of LT by the maximum likelihood method with 1000 bootstrap replicates. Bootstrap values above 50% are shown. For all PyVs included in the tree, GenBank accession numbers, full names, and genera of are shown. TbelPyV1 detected in this study is indicated in bold with solid triangle. Another shrew PyV, *Tupaia glis* polyomavirus, is indicated in bold

The putative LT, ST, VP1, and VP2 aa sequences of TbelPyV1 were aligned with those of related PyVs. With aa identities of 64–77.5% and 61.5–76.4%, LT, ST, VP1, and VP2 of TbelPyV1 shared the highest aa sequence identities with the corresponding proteins of 2 primate PyVs, *Human polyomavirus 9* (HPyV9, GenBank no. NC_015150) and *African green monkey polyomavirus* (AGMPyV, GenBank no. NC_004763), respectively (Table 4). To further determine the genetic relationship between TbelPyV1 and the other PyVs, phylogenetic tree was constructed based on the alignment of LT. In the tree, TbelPyV1 fell within the clade of genus *Alphapolyomavirus* and clustered with HPyV9 and AGMPyV (Fig. 3).

## Discussion

Both PVs and PyVs have double-stranded circular DNA genomes and share many structural features. Because of that, PVs and PyVs were formerly classified into the same family of *Papovaviridae* [33]. However, the genomes of PVs and PyVs are quite different in size and organization, and contain no major sequence homology to each other. In addition, PVs and PyVs show other different biological properties, like PV transcription is unidirectional, but PyV transcription is bidirectional. Because of these differences, PVs and PyVs were classified in the year 2000 into two different families, *Papillomaviridae* and *Polyomaviridae* [8, 34].

Recently, the *Papillomaviridae* family has been divided into 2 subfamilies, *Firstpapillomavirinae* and *Secondpapillomavirinae*, due to the extension of host range and phylogenetic relationships of PVs [34, 35]. The *Firstpapillomavirinae* subfamily consists of 53 genera including PVs infecting mammals, reptiles, and birds. The *Secondpapillomavirinae* subfamily has only one *Alefpapillomavirus* genus including PVs infecting fishes. The L1 is the most conserved gene among PVs. To distinguish genus, less than 60% sequence identity of the pairwise alignments of the L1 genes was used as the criterion. Practically, visual inspection of phylogenetic tree was also considered [36].

The L1 gene of TbelPV1 showed the highest similarity to that of viruses in genera of *Mupapillomavirus*, *Kappapapillomavirus* and *Lambdapapillomavirus*. However, TbelPV1 does not cluster with any of the above 3 genera, but branches off at the root of the common branch of the *Mupapillomavirus* and *Kappapapillomavirus* genera (Fig. 2). As for the TbelPV2, though it was clustered with genus *Iotapapillomavirus* according to the phylogenetic tree, it showed a long evolutionary distance with MnPV1 in *Iotapapillomavirus*. Due to these facts, we suggest that TbelPV1 and TbelPV2 could be considered as the representative species of two novel genera in *Firstpapillomavirinae*.

PVs are highly species-specific and have a specific tropism for squamous epithelial cells [37], and previous evidence showed that a large spectrum of different PVs exhibit skin tropism. The majority of PV infections are subclinical, while certain PVs are associated with an increasing number of squamous cell carcinomas at specific sites, for example, HPV types 16, 18, 31, 33, 35 [7, 38]. By phylogenetic analysis, TbelPV1 clustered with members of genera *Mupapillomavirus* and *Kappapapillomavirus* which indicates a common ancestor. In *Mupapillomavirus* genus, HPV1 is the causative agent of human deep palmo-plantar warts, and HPV63 causes punctate keratotic lesions of the foot [39, 40]. In *Kappapapillomavirus* genus, *Rabbit oral papillomavirus 1* causes benign epithelial infections in the oral cavity and *Cottontail rabbit papillomavirus 2* infects various sites of skin of cottontail rabbit and causes warts [4, 41, 42]. The TbelPV2 showed close genetic relationship to MnPV1 which infects different skin sites of *Mastomys natalensis* and induces tumors [43]. In this study, the TbelPV1 and TbelPV2 were only detected in oral swabs, which indicates that both viruses exhibit oral epithelium tropism. Whether these viruses could cause any lesions needs further investigation.

The *Polyomaviridae* family contains 4 genera *Alpha*-, *Beta-*, *Delta-*, and *Gammapolyomavirus*. The novel species of PyV infecting fish have not yet been assigned to a genus [8]. Polyomavirus-like sequences that did not exhibit the typical genome organization of PyVs, have also been found in arthropods, which indicates that PyVs infect or previously infected invertebrates, probably indicating an ancient evolutionary history [44]. In order to define PyV species, the ICTV established 5 criteria [8]. The TbelPyV1 characterized in this study showed 60.8% nt identity to the most closely related species HPyV9 in the LT coding sequence, which is much less than the criterion (< 85%). The TbelPyV1 genome displays an organization typical for PyVs, and the natural host has been determined. In the phylogenetic tree, TbelPyV1 clustered with other PyVs in the genus *Alphapolyomavirus*, which suggests that TbelPyV1 is a novel species in this genus.

For most mammalian PyVs, the target cells for initial entry, and the exact routes of infection and transmission, are unclear. Previous studies indicate MCPyV, HPyV6, and HPyV7 may initially infect skin and lead to skin-to-skin contact transmission [45]. The MWPyV, HPyV10, STLPyV, HPyV11, and HPyV12 have been detected in the gastrointestinal tract which suggests a fecal-oral transmission route [46]. The JCPyV, BKPyV, KIPyV, and WUPyV are frequently detected in tonsillar tissue and respiratory aspirates, suggesting a route of respiratory tract transmission. In addition, the JCPyV has been found in lymphoid tissues including bone marrow and spleen, BKPyV in lung and salivary glands, WUPyV and KIPyV in plasma and urine [47, 48]. These findings suggest that PyVs can infect various tissues and use diverse transmission routes. TbelPyV1 was most closely related to the HPyV9 and a PyV derived from African green monkey (*Chlorocebus aethiops*) (AGMPyV) (Fig. 3). AGMPyV is lymphotropic and HPyV9 was found in the serum of a kidney transplant patient under immunosuppressive treatment [49]. Here, TbelPyV1 was detected in oral and spleen samples with high rates, which indicates that oral tissues like tonsil and spleen are the major sites of infection for TbelPyV1. However, we did not perform pathological examination due to limited tissue samples. Therefore, the pathogenicity of TbelPyV1 remained unclear.

PVs and PyVs evolve remarkably slowly (1.95 × 10^− 8^ for PVs and 8 × 10^− 9^ for PyVs, measured in nucleotide substitutions per site per year) and are assumed to be ancient viruses that have co-evolved with their host species at least over half a billion years [44, 50]. These characteristics make PVs and PyVs fascinating candidates for development of viral evolutionary and virus-host co-evolutionary models. In addition, phylogenetic analysis based on genomic level showed that Scandentia are most closely related to primates and may be the evolutionary transition from insectivores to primates [17, 51]. Our first description of 2 novel PVs and a novel PyV in tree shrews indicates that tree shrews may carry diverse PVs and PyVs. The novel genomes of PVs and PyV characterized in this study, together with the additional PVs and PyVs discovered in tree shrews in the future, would promote the study of viral evolution and virus-host co-evolution, and might shed light on the origin of PVs and PyVs of primates.

## Conclusion

Two novel papillomaviruses (TbelPV1 and TbelPV2), and one novel polyomavirus 1 (TbelPyV1) were detected in Chinese tree shrews, and their genomes were characterized. Phylogenetic analysis based on the L1 indicated that TbelPV1 and TbelPV2 represent members of two novel PV genera. Phylogenetic analysis based on the LT indicated that the TbelPyV1 represents a new species within genus *Alphapolyomavirus*. The epidemiological study showed that TbelPV1 and TbelPV2 were prevalent in the oral cavity, while TbelPyV1 was prevalent in the oral cavity and spleen. Whether these viruses cause disease in tree shrews needs further investigation.

## Additional file

Additional file 1: **Table S1.** Primers used for detection and sequencing of TbelPV1, TbelPV2, and TbelPyV1 in tree shrew. (DOCX 17 kb)

## Abbreviations

BLAST: Basic Local Alignment Search Tool
HPB: Human papillomavirus HPV
ICTV: The International Committee on Taxonomy of Viruses
LCR: Long control region
LT: Large T antigen
ORF: Open reading frame
PCR: Polymerase chain reaction
PV: Papillomavirus
PyV: Polyomavirus
ST: Small T antigen
TbelPV1: *Tupaia belangeri chinensis* papillomavirus 1
TbelPV2: *Tupaia belangeri chinensis* papillomavirus 2
TbelPyV1: *Tupaia belangeri chinensis* polyomavirus 1
URR: Upstream regulatory region
