# Supplementary material for: Detection and genome characterization of two novel papillomaviruses and a novel polyomavirus in tree shrew (Tupaia belangeri chinensis) in China
Source: Virol J. 2019 Mar 18;16:35. doi: 10.1186/s12985-019-1141-9 (PMC6423848; doi:10.1186/s12985-019-1141-9)
Supplement: Supplementary file 1 — Table S1. Primers used for detection and sequencing of TbelPV1, TbelPV2, and TbelPyV1 in tree shrew. (DOCX 17 kb) [file 12985_2019_1141_MOESM1_ESM.docx]

Table S1. Primers used for detection and sequencing of TbelPV1, TbelPV2, and TbelPyV1 in tree shrew.

| **primers** | **5' to 3'** | **replicon** |
| --- | --- | --- |
| PV1F | GACATTATAGGCGTGCAGAAATGAATG | 313bp |
| PV1R | GCCAGGGGTTGCAGCCAAAAATGGCTC |  |
| PV2F | CAGGCATTATAAGCGGGCAGAAATG | 347bp |
| PV2R | TGGCATCATCTAGCAGTGCCACTTTCG |  |
| PyVF | GCTTGGCTCTGGGTGGGCTGCTCCTC | 321bp |
| PyVR | GGATGTATCTCTGTTGAACATCTGG |  |
| PV1F1 | CATGACTTCTAGGTCACTCACTAATG | 2537bp |
| PV1R1 | CCTCGCCTTCTATCTCGCCAACACAC |  |
| PV1F2 | CAGAAGACATATATCAGCACTGTAAAC | 3189 |
| PV1R2 | TATTGAGACACACAAGCAGACAAACAG |  |
| PV1F3 | CATGAGCATGCTGAAAGTCCTAAAGG | 1794 |
| PV1R3 | TACACCAGCACTGCCGTACTGCAGGA |  |
| PV2F1 | CAAGTGGAACAGCATGACCGCGATC | 1401bp |
| PV2R1 | CAAGTGGAACAGCATGACCGCGATC |  |
| PV2F2 | CTCAGAAGCAGACTAACAATGTTCC | 3041bp |
| PV2R2 | GAAAGATGAGTAGTCCACCACAATGC |  |
| PV2F3 | ATGTCCAATGAGCTCTATGGCAATAAC | 560bp |
| PV2R3 | GACTGTATGTAGCGGTACTTGTCTTC |  |
| PyVF1 | GGCAAGAATGCGTGCCATGTCCTAAGAA | 2922bp |
| PyVR1 | AAGGCCTAGAACAGAGGATTCTACCTG |  |
| PyVF2 | TACTAGGCCCAGGTTCTTCCTCAGAAC | 2422bp |
| PyVR2 | GTTGGTCATCATGTTTGCCTTGGGCAG |  |
